# Difficulties with Pitch Discrimination Influences Pitch Memory Performance: Evidence from Congenital Amusia

**DOI:** 10.1371/journal.pone.0079216

**Published:** 2013-10-25

**Authors:** Cunmei Jiang, Vanessa K. Lim, Hang Wang, Jeff P. Hamm

**Affiliations:** 1 Music College, Shanghai Normal University, Shanghai, China; 2 Cognitive Neuroscience Research Group, University of Auckland, Auckland, New Zealand; Northwestern University, United States of America

## Abstract

Music processing is influenced by pitch perception and memory. Additionally these features interact, with pitch memory performance decreasing as the perceived distance between two pitches decreases. This study examined whether or not the difficulty of pitch discrimination influences pitch retention by testing individuals with congenital amusia. Pitch discrimination difficulty was equated by determining an individual’s threshold with a two down one up staircase procedure and using this to create conditions where two pitches (the standard and the comparison tones) differed by 1x, 2x, and 3x the threshold setting. For comparison with the literature a condition that employed a constant pitch difference of four semitones was also included. The results showed that pitch memory performance improved as the discrimination between the standard and the comparison tones was made easier for both amusic and control groups, and more importantly, that amusics did not show any pitch retention deficits when the discrimination difficulty was equated. In contrast, consistent with previous literature, amusics performed worse than controls when the physical pitch distance was held constant at four semitones. This impaired performance has been interpreted as evidence for pitch memory impairment in the past. However, employing a constant pitch distance always makes the difference closer to the discrimination threshold for the amusic group than for the control group. Therefore, reduced performance in this condition may simply reflect differences in the perceptual difficulty of the discrimination. The findings indicate the importance of equating the discrimination difficulty when investigating memory.

## Introduction

Pitch perception and pitch memory are interwoven in the processing of music [1,2]. In order to discriminate/identify pitch changes in a sequence of pitches it is necessary to form a memory of the pitch events heard. This pitch memory trace would be dependent on the initial perception of the pitches. Musical expertise influences pitch memory with musicians outperforming nonmusicians on pitch memory tasks [3,4]. Memory for pitch might be stored in a system that is associated with a pitch perceptual representation system [5,6].

It has been reported since the 1960s that phonological similarity has a negative impact on verbal working memory [7–10]. This is also the case for pitch memory where pitch proximity impacts upon memory performance. By manipulating the pitch distance between a standard tone and the intervening tone(s) in a memory task, memory performance was worse for small pitch distances than for large pitch distances [11]. Similarly, when the pitch distance between the pitches of notes within to-be-remembered sequences was manipulated, nonmusicians showed a significant negative effect of pitch proximity, while musicians showed no effect of pitch proximity for musical sequences [4]. This interaction between pitch proximity and expertise has been demonstrated previously [12]. Differences between musicians and nonmusicians in the pitch discrimination difficulty of the tasks may account for the interaction between pitch proximity and expertise. In other words, the pitch-proximal tones may sound more similar for the nonmusicians than for the musicians because of the differences in pitch perception between musicians and nonmusicians. 

In this investigation, we examined whether or not pitch discrimination difficulty influences pitch memory performance by testing individuals with congenital amusia, a neuro-developmental disorder of pitch processing. Individuals with congenital amusia (hereafter amusia) show deficits in discriminating fine-grained pitch changes [13–16], pitch contour [13,17], and pitch direction [13,18,19]. These musical pitch deficits extend to speech processing with amusia [17,18,20–27]. 

 In addition to the pitch discrimination deficits, some studies have investigated the potential for a pitch specific memory deficit to be associated with amusia [28–32], and furthermore another has examined the possibility of timbre memory deficits [33]. Among these studies, four studies employ the same stimuli for both the controls and the amusics [28,29,31,32] while in one study the stimuli were adjusted for amusics with high pitch discrimination thresholds [30]. However, even in this last study, a common set of stimuli were employed for the majority of amusics, and all controls. 

The use of the same stimuli for both groups complicates the interpretation of the data from pitch memory studies involving amusics because the tones are less discriminable for amusics than for controls, even when the pitches are quite far apart [15,28]. If both amusics and controls are required to discriminate differences in pitch using stimuli separated by the same interval, then the perceptual discrimination required is harder for the amusics than for the controls. Therefore, unless the stimuli for both groups are equated at the individual level for perceptual difficulty and familiarity, then the finding that the amusic group performs more poorly on the memory task does not necessarily lead to the conclusion that there is impairment in a pitch memory system. Rather, such a finding may simply reflect the amusic group’s increased difficulty with the stimulus discrimination.  Put more simply, such a finding may simply reflect increased task difficulty.

In this investigation, we examined pitch retention in amusia when stimuli were equated on an individual basis for the perceptual difficulty of the discrimination. A standard tone comparison paradigm developed by Deutsch [34] was employed in the current memory study. We included two retention tasks: a same/different pitch discrimination task and a pitch direction identification task. Moreover, a simple two down one up staircase procedure was used to equate the discrimination difficulty at a 70.7% accuracy level [35], which for an unbiased observer using a difference strategy would translate to a d’ value of 1.76 [36]. This discrimination threshold value can then be used to create different conditions of similar difficulty.  Because the intervals between the resulting tones are generally not tonal, and do not correspond to semitones, the changes in pitch will be unfamiliar to both amusics and controls. If amusics have normal pitch memory but pitch memory task performance is influenced by the perceptual difficulty of the discrimination, then performance for both amusics and controls should improve at the same rate as the discrimination is made easier. However, when the same stimuli are employed for both groups, it is expected that the amusics’ performance will appear to show a pitch memory deficit even when the stimuli are set well above the amusic group’s discrimination threshold. As with a previous study [32], a constant difference of four semitones was employed in the current study.

## Methods

### Participants

Participants were recruited by means of advertisements in the bulletin board system of universities in Shanghai. Their music abilities were tested on the Montreal Battery of Evaluation of Amusia (MBEA) [37]. A detailed questionnaire was conducted to get further information about the participants. Fourteen amusics and 14 controls were tested in the current study. None of them reported a history of neurological, psychiatric diseases, or any learning or memory problems with their university studies. Among all participants, two amusics received extracurricular musical training: one for six months, and other one for three years. All but two participants (one amusic and one control participants, respectively) were right-handed as assessed by the Edinburgh Handedness Inventory [38]. The demographic characteristics and MBEA scores of the participants are shown in Table 1. Ethical approval was attained from Shanghai Normal University in China, and written informed consents were obtained from all of the participants before testing.

**Table 1:** Participants’ demographic characteristics and the MBEA scores.

Demographic Characteristics	Amusic (n=14)	Control (n=14)	*t*-test
Mean age (SD)	27(7.34)	27(7.64)	NS
Gender	7M,7F	7M,7F	
Years education (SD)	17(1.7)	17(1.2)	NS
Global score of MBEA (SD)	20(1.7)	28(0.8)	*p* < .001
Scale subtest (SD)	17 (3.0)	28 (1.5)	*p* < .001
Contour subtest (SD)	19 (2.8)	28 (1.7)	*p* < .001
Interval subtest (SD)	17 (2.3)	28 (1.5)	*p* < .001
Rhythm subtest (SD)	23 (3.1)	28 (1.8)	*p* < .001
Meter subtest (SD)	20 (6.0)	27 (2.2)	*p* = .001
Memory subtest (SD)	21 (4.2)	29(1.2)	*p* < .001

Note: F = female; M = male.

### Pretests: Psychophysical measures of pitch perception

#### Method and procedure

As noted above, the purpose of the pretest was to determine each participant’s perceptual 70.7% accuracy threshold [35]. This ensured that the pitch distance used in the subsequent memory tasks was not below the chance perceptual threshold for any given participant. This also enabled the equating of the discrimination difficulty of the subsequent retention tasks. Pitch change detection and pitch direction discrimination threshold tests were included in the pretests. To avoid a perceptual direction bias, each test contained two tasks. In one task the low tone was kept constant as the high tone was changed, while in the other task the high tone was held constant and the low tone was changed. A two-alternative forced choice AXB procedure was used in the two threshold tests since the AXB paradigm is regarded as a procedure demanding reduced short-term memory [39,40]. 

**Figure 1:**
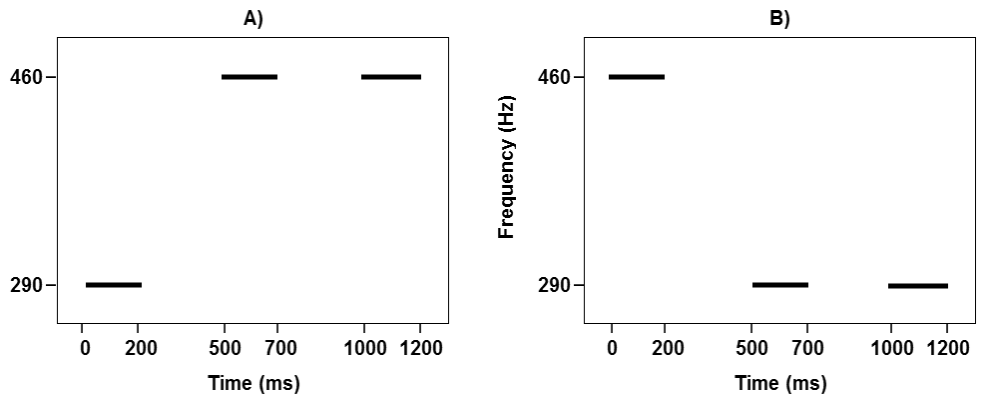
Illustrations of the pitch change detection threshold tasks. A) Low tone held constant while high tone changes. B) High tone held constant while low tone changes.

For the pitch change detection threshold test, the participants were presented with three pure tones: A, X, and B. The second tone (X) was identical to either the first one (A), or the last one (B) as illustrated in Figure 1. Among the two tasks of the pitch change detection threshold test, the frequencies of the first tones (A) were 290 Hz and 460.4 Hz, respectively, and correspondingly the last tones (B) either varied from 290.3 Hz to 460.4 Hz, or from 459.8 Hz to 290 Hz in frequency. Each pure tone was 200 ms in duration, gated with 10 ms onset and offset amplitude ramps, and was separated by a silent interval of 300 ms. Participants were required to determine whether the second tone was identical in pitch to the first or last tone. 

**Figure 2:**
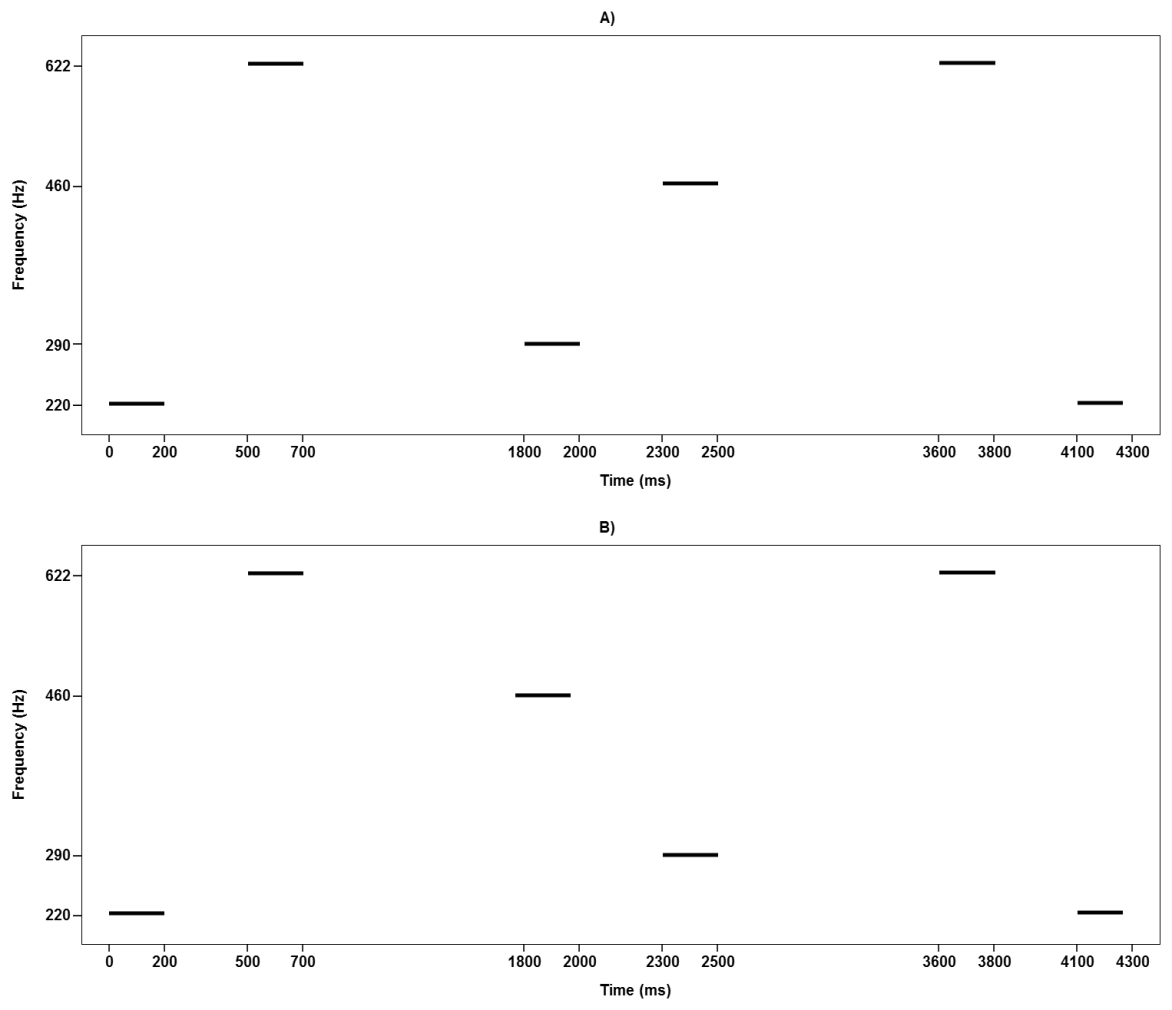
Illustrations of the pitch direction discrimination threshold tasks. A) Low tone held constant while high tone changes. B) High tone held constant while low tone changes.

For the pitch direction discrimination threshold test, the only difference from the pitch change detection test is that each of the three parts of A, X, and B in the AXB paradigm contained a two-tone pair. The two-tone pairs in the first (A) and the third (B) parts were always kept constant, while the two-tone pair in the second part (X) was adjusted. More specifically, the first tone and the second tone in the first part (A) were always 220 Hz and 622.3 Hz in frequency (low-high), while the first tone and the second tone in the third part (B) were always 622.3 Hz and 220 Hz in frequency (high-low). Among the two tasks of the pitch direction discrimination threshold test, by analogue, the frequencies of the first tones in the second part (X) were 290 Hz and 460.4 Hz, respectively, and correspondingly the second tones in the second part (X) either varied from 290.3 Hz to 460.4 Hz or from 459.8 Hz to 290 Hz in frequency. The three parts in the AXB paradigm were separated by a silent interval of 1100 ms as illustrated in Figure 2. Participants were told that the two-tone pairs in the first (A) and the third (B) parts were kept constant, respectively, and required to indicate whether the two-tone pairs in the second part (X) moved in the same direction as those in the first (A) or third part (B).

Experimental trials were presented with an adaptive staircase procedure using the software package E-Prime. The initial pitch difference was four semitones and was varied in terms of a “two- down, one- up” staircase procedure, where the pitch distance increases when an incorrect response is given, and decreases only after two consecutive correct responses. We used a step size of one semitone for the reversals one through four, a step size of 0.1 semitones for the reversals five through eight, and a step size of 0.02 semitones for the reversals nine through sixteen. The procedure terminates after 16 reversals, and the threshold was computed based on the average the last six reversals. The number of steps to reach threshold did not differ between the groups for either the pitch change (*t*(26) = 1.46 , p > 0.05, mean number of steps 63.6 vs 53.5, amusics and controls, respectively) or the pitch direction (*t*(26) = 0.48, *p* > 0.05; mean number of steps 71.3 vs 66.7, amusics and controls, respectively) staircase. In addition, threshold estimates remained stable after turnaround 9 for both groups in both tasks. Participants were given six practice trials for pitch change detection and ten practice trials for pitch direction discrimination, respectively. Among the practice trials, the pitch difference between the first and the last tones for pitch detection, and between the first and the second tones in the second part (X) for pitch direction ranged from six to ten semitones. Feedback was provided only for the practice trials. The four threshold tasks were presented in a counterbalanced order across participants.

#### Results

**Figure 3:**
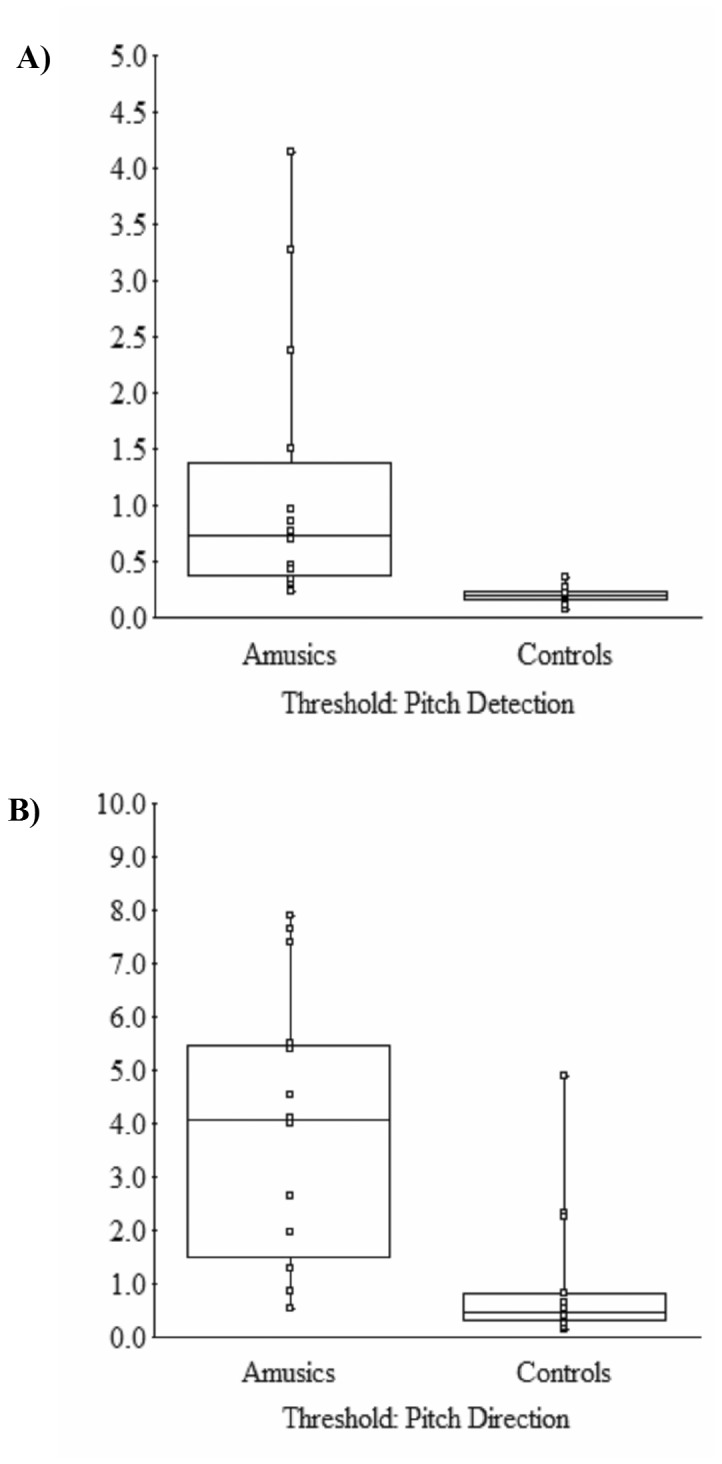
Box and Whiskers plot showing individual’s discrimination thresholds for the amusic and control groups. A) Pitch change detection. B) Pitch direction discrimination. The box indicates the 25, 50, and 75% cut offs, with the whiskers spanning to the maximum and minimum values.

The thresholds for the two tasks in the pitch change detection and pitch direction tests were averaged, respectively. Figure 3 shows box and whisker plots of the individuals’ averaged thresholds for both groups from the A) pitch change detection and B) pitch direction discrimination. The amusic participants had higher thresholds than the controls for both of pitch change detection [1.18 vs 0.20 semitones, respectively; *t*(13.13) = 2.99, *p* < 0.05; degrees of freedom are adjusted because of unequal variance between the groups; log transformation of the threshold data leads to the same results] and pitch direction discrimination [3.90 vs 1.01 semitones, amusics vs controls, respectively; *t*(19.21) = 3.67, *p* < 0.05]. The ratio of these threshholds (pitch direction / direct discrimination) did not differ between the groups [*t*(17.97) = -0.45, *p* > 0.05, mean threshold ratios of 5.14 and 6.24 for the amusic and control group, respectively]. It can be observed from Figure 3 that there were overlaps in thresholds between the two groups in both tasks. Overall, 10 out of 14 amusics had pitch detection thresholds below one semitone with 4 amusics scoring detection thresholds over one semitone. The pitch discrimination threshold was correlated with the pitch direction discrimination threshold for the amusic group [*r*(12) = 0.62, *p* < 0.05], but not for the controls [*r*(12) = 0.26, *p* > 0.05]. It is worth noting that nothing changes if the data of the 4 amusics with thresholds over 1 semitone are dropped**.**

The data were consistent with previous studies that have found that amusics have higher pitch change detection thresholds than controls [13,18,30], and replicates the standard finding of a pitch discrimination deficits in amusia. For pitch direction discrimination, given that pitch glide is rarely applied to musical works, the current study required participants to discriminate the pitch change direction between two separate pitches to examine pitch direction perception, and did not employ pitch glide as previous studies did [13,32,41]. Consistent with previous studies [13,18,41], the current data showed that amusics exhibited higher pitch direction thresholds than the controls, although this is not always found [32]. This discrepancy between the study by Williamson and Stewart [32] and the current study may be primarily due to the difference of experimental stimuli (i.e., pitch glide used in the former, and segmented tone pair used in the current study).

As noted above, to examine the effects of discrimination difficulty on pitch memory, the pitch distance used in the subsequent memory tasks contained three conditions defined by the magnitude of the difference between the tones, either one, two, or three times the 70.7% discrimination thresholds [35]. Moreover, to facilitate comparisons with the literature, a constant four semitone pitch distance was also included [32], resulting in four pitch distance conditions. 

### Single-tone pitch change detection and pitch direction discrimination

#### Stimuli

Two tasks were included in the current study: a single-tone pitch retention task (same/different discrimination) and a pitch direction retention task (direction identification). In each task, participants were presented with two pure tones: a standard tone and a comparison tone, separated by a period of silence or four intervening tones. The intervening tones were four different pure tones that did not include either the standard tone or the comparison tone and each of intervening tones was at least two semitones higher or lower than the standard tone [42–44] , with the first intervening tones being a minimum of six semitones away from the standard tone. Each of the intervening tones was a minimum of six semitones away from the previous tone, which is close to two semitones larger than the highest threshold of all participants measured in the pitch detection test. 

Each tone was 70 dB lasted 200 ms in duration, gated with 10 ms onset and offset linear amplitude ramps. In the silence interval, the standard and comparison tone were separated by a silent interval of four seconds. In the distractor condition the standard tone and the four intervening tones were separated by intervals of 300 ms, except for a two-second pause between the last intervening tone and the comparison tone. There were 48 pairs for the standard and comparison tone. For the single-tone pitch retention, half of the pairs were identical with the other half being different. For the pitch direction retention, among 48 pairs, the comparison tones in the half of pairs were higher than the standard tones, while the comparison tones were lower than the standard tones for the other half of pairs. When different, the physical pitch distance between the standard and comparison tones varied between participants based upon their relevant threshold settings from the pretests to produce three perceptually matched conditions of 1x, 2x, and 3x threshold. In addition, a constant difference condition of four semitones (physically matched but perceptually varying) was employed to determine if not basing stimuli upon thresholds would lead to similar conclusions. Each pair was employed in combination with the two interval conditions (silence and intervening tones), resulting in total of 192 trials in the pitch retention and pitch direction retention tasks, respectively.

#### Procedure

There were five blocks for each task. Within each block, the trials were presented in a pseudo-randomized order with the constraint that two consecutive trials never be identical different pairs. Participants were told that they would hear two tones: a standard tone and a comparison tone, which would be separated either by silence or four intervening tones. Participants were told to ignore the four intervening tones when they occurred. For the single-tone pitch retention task participants were told to listen carefully in order to make a same/different judgment on the standard and the comparison tones. For the pitch direction task, participants were required to indicate whether the comparison tone was higher or lower than the standard tone. The two tasks were presented in a counterbalanced order across the participants. There were six practice trials with feedback in each task. No feedback was given during the experimental trials. All stimuli were presented binaurally through Philips SHM1900 headphones in a soundproof room.

## Results

Performance on both tasks was measured by the sensitivity index d’. In the single-tone pitch retention task a hit was defined as responding different to a different pair, while a false alarm was defined as responding different to an identical pair. In the pitch direction retention task a hit was defined as responding downward when the C tone was lower than the S tone, while a false alarm was defined as responding upward when the C tone was lower than the S tone. To avoid the biasing effect of extreme proportions on values of d’ the log-linear correction recommended by Hautus [45] was used, d’ was calculated as for a same/different task based upon a differencing model in the single-tone pitch retention, and calculated as for a two alternative forced choice task in pitch direction retention [36]. 

### Single-tone pitch change detection analysis

**Figure 4:**
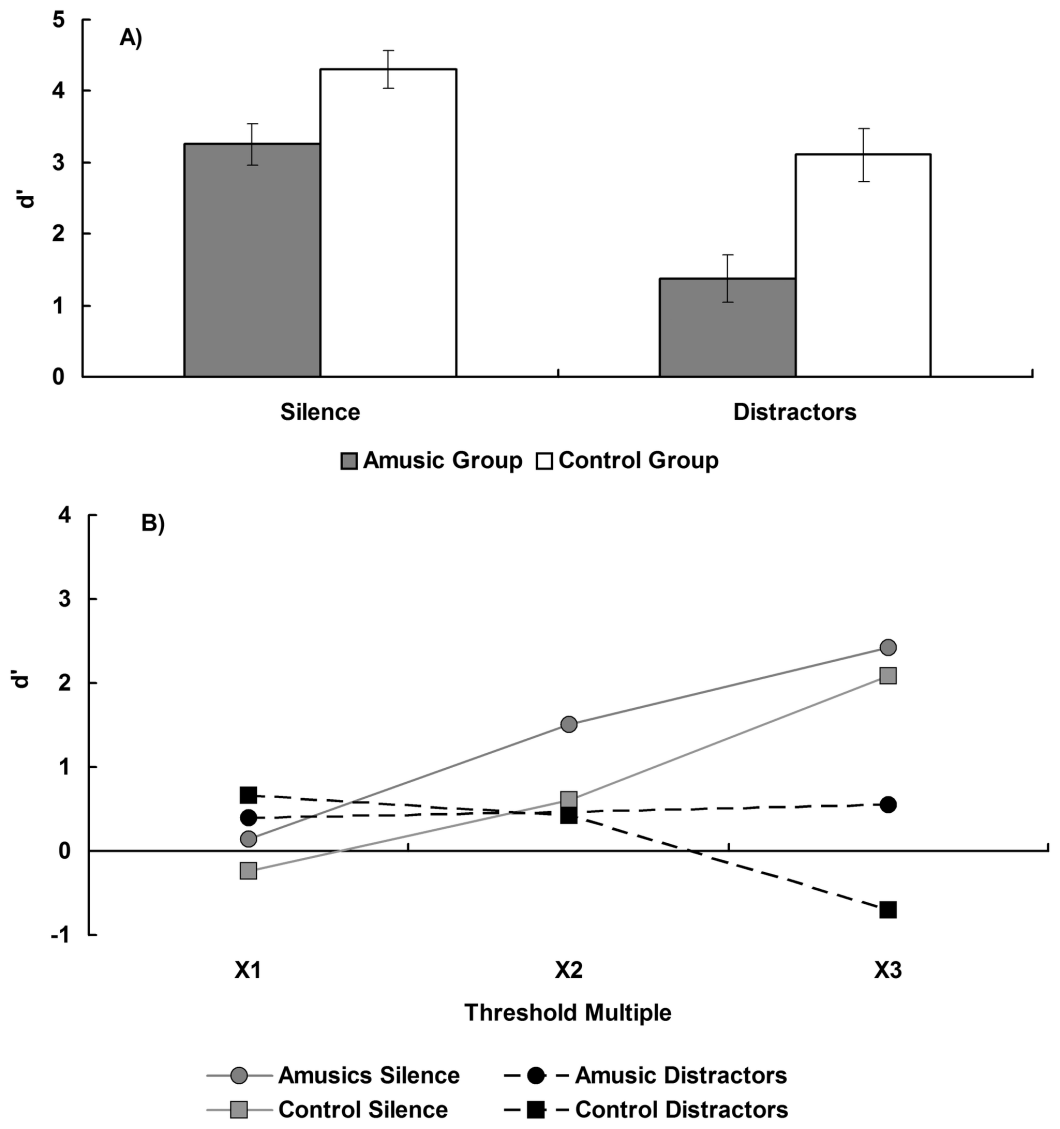
Sensitivity index (d’) of single-tone pitch change detection for the two groups. A) Different trials employ a constant four semitones change with a silent interval and distractors, respectively. Error bars indicate ±1 standard error. B) As a function of an individual’s threshold for both silent and intervening tone conditions.

The d’ data from the four semitone condition were analysed in a two-way, mixed factor ANOVA, with interval content (silence/distractors) as a within subjects factor and group (amusic/controls) as a between groups factor. This resulted in a main effect of group [F(1,26) = 13.26, p < 0.05] with the controls outperforming the amusics (d’ = 3.71 vs 2.32, respectively). There was a main effect of interval content [F(1,26) = 42.96, p < 0.05], with the intervening tones interfering with the pitch retention (d=2.24 vs 3.78 for distractors and silence, respectively). There was no interaction between group and interval condition, suggesting the effect of the intervening tones was of a similar magnitude for both groups [F(1,26) = 2.10, p > 0.05]. These data can be seen in Figure 4A and indicate that when a constant pitch distance (four semitones) is used amusics have impaired performance on the pitch retention task compared to controls, and both groups are similarly impaired by the presence of intervening tones.

The d’ values for the threshold matched pitch distance condition was analysed in a mixed factor, three-way ANOVA, with threshold setting (1x, 2x, 3x pitch change detection threshold) and interval content (silence/distractors) as within subjects factors and group (amusic/controls) as a between subjects factor and can be seen in Figure 4B. Performance at the 1x threshold level in silence was below the d’ value of 1.76 that the staircase procedure should target for both groups [t(13) = -4.39, and t(13) = -6.15, both p < 0.05, for amusics and controls, respectively]. This is consistent with signal loss occurring due to the increase in retention time between the S and C tones during the memory test relative to the staircase procedure. The threshold factor was investigated for trends with planned contrasts to test for linear and quadratic components, and non-significant trends will not be reported and can be assumed to be associated with p > 0.05. This resulted in a main effect of interval content [F(1,26) = 11.42, p < 0.05], with the interval of intervening tones resulting in overall worse performance than the silent interval (d’= 0.30 vs 1.09, respectively), and a main effect of threshold setting [F(2,52) = 9.84, p < 0.05], which was due to a significant linear trend [F(1,26) = 20.20, p < 0.05]. There was a significant interaction between interval content and threshold setting [F(2,52) = 25.55, p < 0.05], which was primarily due to an interaction in the linear trend components [F(1,26) = 55.76, p < 0.05]. There was no main effect of group (F(1,26) = 1.08, p > 0.05), nor did group interact with either interval content [F(1,26) = 0.18, p > 0.05] or threshold setting [F(2,52) = 1.93, p > 0.05] at the omnibus test level, although the interaction between group and the planned linear trends was significant [F(1,26) = 4.28, p < 0.05]. Similarly, the omnibus test of the three way interaction between threshold setting, interval content, and group was not significant [F(2,52) = 2.84, p > 0.05], however the 3-way interaction in the linear trend components was at the criterion level of significance [F(1,26) = 4.06, p = 0.05].

To examine the potential three way interaction in more detail, a simple effects analysis was performed examining the factors threshold and group at each level of interval content separately. These two-way mixed factor ANOVAs revealed that when the interval was silent, there was a main effect of threshold setting [F(2,52) = 42.79, p < 0.05], which was due to a linear trend [F(1,26) = 92.54, p < 0.05], but no difference between the groups [F(1,26) = 1.19, p > 0.05] nor was there an interaction between threshold setting and group [F(2,52) = 0.82, p > 0.05], and of specific interest, the test for the interaction between group and the linear trend was not significant [F(1,26) = 0.004, p > 0.05]. This indicates that when the interval was silent, single-tone pitch memory performance did not differ between the groups when the perpetual difficulty was equated. Additionally, memory performance improved as the discrimination was made easier for both groups.

When the interval was filled with intervening tones, there was no main effect of group [F(1,26) = 0.52, p> 0.05] nor was there a main effect of threshold setting [F(2,52) = 2.24, p > 0.05], although the linear trend was significant [F(1,26) = 4.29, p < 0.05]. When performance was collapsed over the threshold values and compared against chance performance (d’ = 0), neither group performed above chance [d’ = 0; t(13) = 1.19, and t(13) = 0.49, both p > 0.05, means 0.47 and 0.13, for the amusic and control groups, respectively]. However, the interaction between threshold and group was significant [F(2,52) = 3.44, p < 0.05], and this was due to an interaction in the linear trend components between the groups [F(1,26) = 6.96, p < 0.05], reflecting the poor performance of the controls at the 3x threshold condition. As performance by the controls was below 0 in the 3x condition, it is presumed that this reflects sampling error around their overall chance level performance and so the interaction between the groups is considered unreliable. As such, the data are interpreted as indicating that when the interval was filled with intervening tones, both groups performed at chance, and neither improved as the pitch distance between the S and C tones was increased.

**Figure 5:**
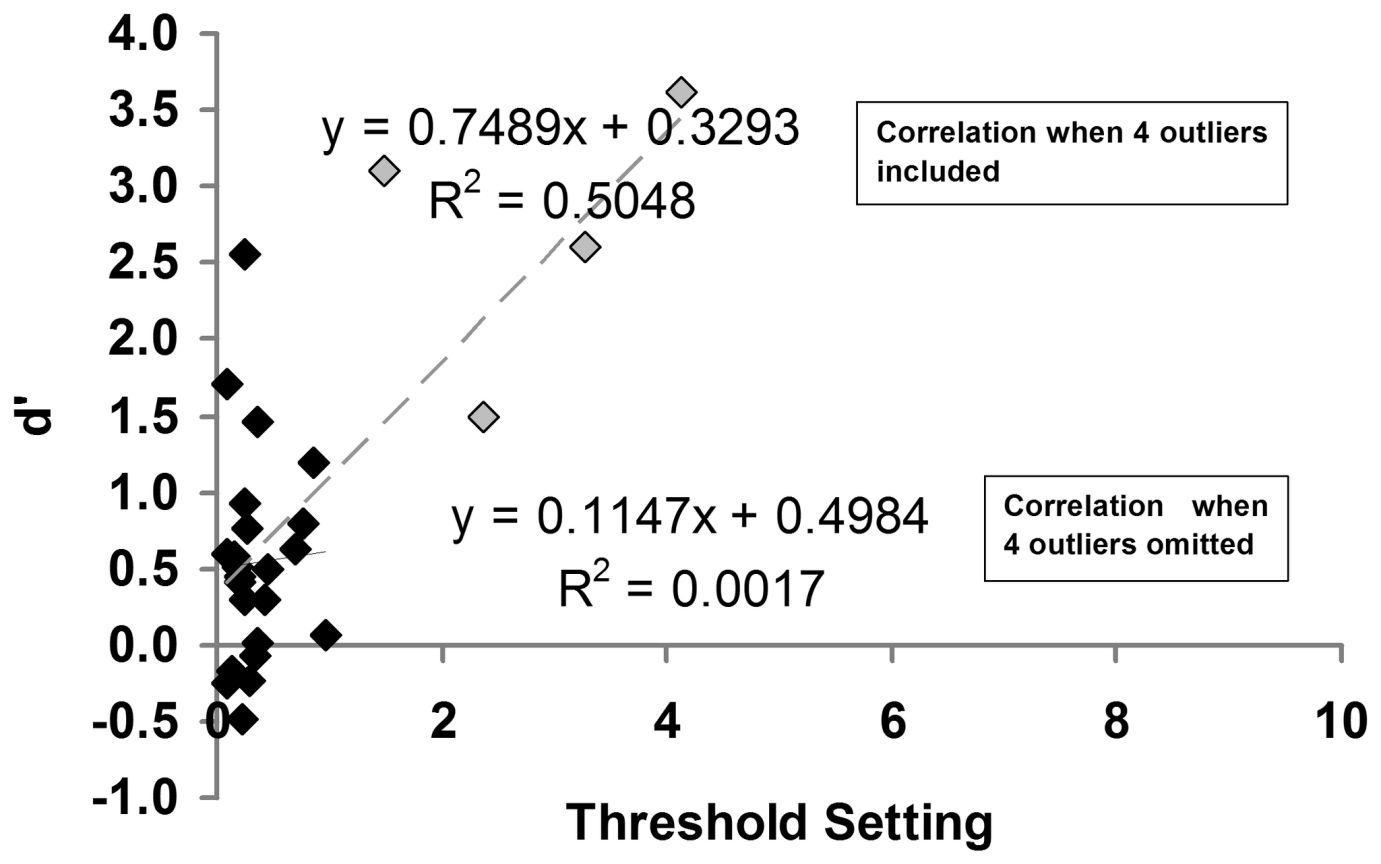
Individual d’ scores on single-tone pitch change detection as a function of the individual’s pitch change threshold. Note: grey squares indicate the outlier data of four amusics who had thresholds above one semitone.

To assess whether pitch memory performance was related to their pitch detection thresholds a single memory score was calculated by collapsing over all pitch change detection conditions except the 3x threshold noise condition, as this produced the negative d’ results for the controls and is thought to reflect sampling error. There was a significant correlation between thresholds and memory performance [r(26) = 0.71, p < 0.05, see Figure 5], however, this appears to be the result of the outlier data of four amusics who had thresholds above one semitone, which when removed, renders the correlation non-significant [r(22) = 0. 04, p > 0.05]. It is noted that the pattern of results for the ANOVA remain unchanged when data from these four amusics were removed.

Overall, the analysis of the single-tone pitch retention task indicates that when the perceptual difficulty, namely difference in the discriminability of the stimuli, is equated between the amusics and controls then performance does not differ between the groups.

### Pitch direction discrimination analysis

**Figure 6:**
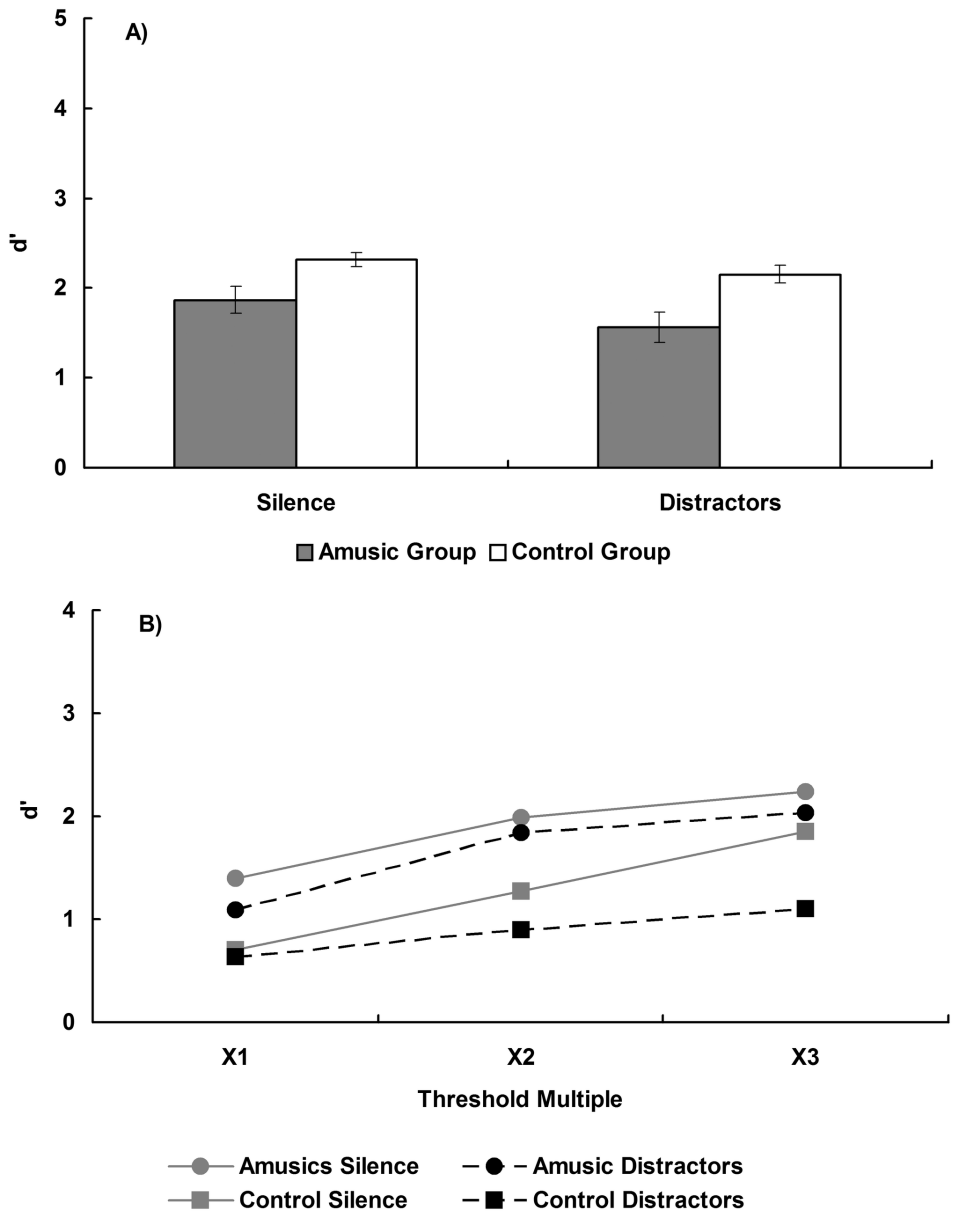
Sensitivity index (d’) of pitch direction discrimination for the two groups. A) Trials employ a constant four semitone difference with a silent interval and distractors, respectively. Error bars indicate ±1 standard error. B) As a function of an individual’s threshold for both silent and distractor conditions.

The d’ data from the four semitone condition were analysed in a two way, mixed factor ANOVA, with interval content (silence/distractors) as a within subjects factor and group (amusic/controls) as a between groups factor. This resulted in a main effect of group [F(1,26) = 9.80, p < 0.05] with the controls outperforming the amusics (d’ = 2.23 vs 1.71, respectively). There was a main effect of interval content [F(1,26) =8.67, p < 0.05], with the intervening tones interfering with the pitch retention (d’=2.09 vs 1.85 for silence vs distractors, respectively). There was no interaction between group and interval condition, suggesting the effect of the intervening tones was of a similar magnitude for both groups [F(1,26) = 0.78, p > 0.05]. These data can be seen in Figure 6A and indicate that when a constant pitch distance is used (four semitones) amusics have impaired performance on the pitch direction task compared to controls, and both groups are similarly impaired by the presence of intervening tones.

The d’ values for the threshold matched condition were analysed in a mixed factor, three-way ANOVA, with threshold setting (1x, 2x, 3x pitch discrimination threshold) and interval content (silence/distractors) as within subjects factors and group (amusic/controls) as a between subjects factor and can be seen in Figure 6B. Performance at the 1x threshold level in silence was below the d’ value of 1.76 that the staircase procedure should target for both groups [t(13) = -2.42, and t(13) = -8.02, both p < 0.05, for amusics and controls, respectively]. This is consistent with signal loss occurring due to the increase in retention time between the S and C tones during the memory test relative to the staircase procedure. The threshold factor was investigated for trends with planned contrasts to test for linear and quadratic components, and non-significant trends will not be reported and can be assumed to be associated with p > 0.05. This resulted in a main effect of interval content [F(1,26) = 15.11, p < 0.05], with performance being higher in the absence of the intervening tones (d’= 1.58 vs 1.26 for silence vs distractors, respectively), a main effect of threshold setting [F(2,52) = 90.54, p < 0.05], which was due to both a significant linear [F(1,26) = 137.90, p < 0.05] and quadratic trend [F(1,26) = 6.05, p < 0.05]. The interaction between interval content and threshold setting did not reach significance [F(2,52) = 3.08, p > 0.05], although the planned contrast testing the interaction in the linear trends was significant [F(1,26) = 8.403, p < 0.05]. There was a main effect of group [F(1,26) = 12.19, p < 0.05] with the amusics outperforming the controls (d’ = 1.76 vs 1.08, amusics vs controls, respectively). This difference between the groups did not interact with either interval content [F(1,26) = 1.28, p > 0.05] or threshold setting [F(2,52) = 2.08, p > 0.05]. However, the three way interaction between threshold setting, interval content, and group was significant [F(2,52) = 5.40, p < 0.05], which was due to a 3-way interaction in the linear trend components [F(1,26) = 15.56, p < 0.05]. 

To examine the 3 way interaction in more detail, a simple effects analysis was performed examining the factors threshold and group at each level of interval content separately. These two-way mixed factor ANOVAs revealed that when the interval was silent, there was a main effect of threshold setting [F(2,52) = 61.19, p < 0.05], which was due to a linear trend [F(1,26) = 160.09, p < 0.05]. There was also a main effect of group [F(1,26) = 14.24, p < 0.05], with the amusics outperforming the controls (d’ = 1.88 vs .128, amusics vs controls, respectively). However, the interaction between threshold setting and group was not significant [F(2,52) = 2.13, p > 0.05]. Moreover the test for the interaction between the linear trends failed to reach significance [F(1,26) = 3.94, p < 0.05]. 

When the interval was filled with distracting tones, there was a main effect of threshold setting [F(2,52) = 35.19, p < 0.05], which was due to a significant linear [F(1,26) = 52.63, p < 0.05] trend. There was also main effect of group [F(1,26) = 9.25, p < 0.05] with the amusics outperforming the controls (d’ = 1.65 vs 0.88, respectively), which interacted with threshold setting [F(2,52) = 5.35, p < 0.05] and which is described by an interaction in the linear trends [F(1,26) = 6.28, p < 0.05], where the controls show a reduced improvement over threshold settings.

**Figure 7:**
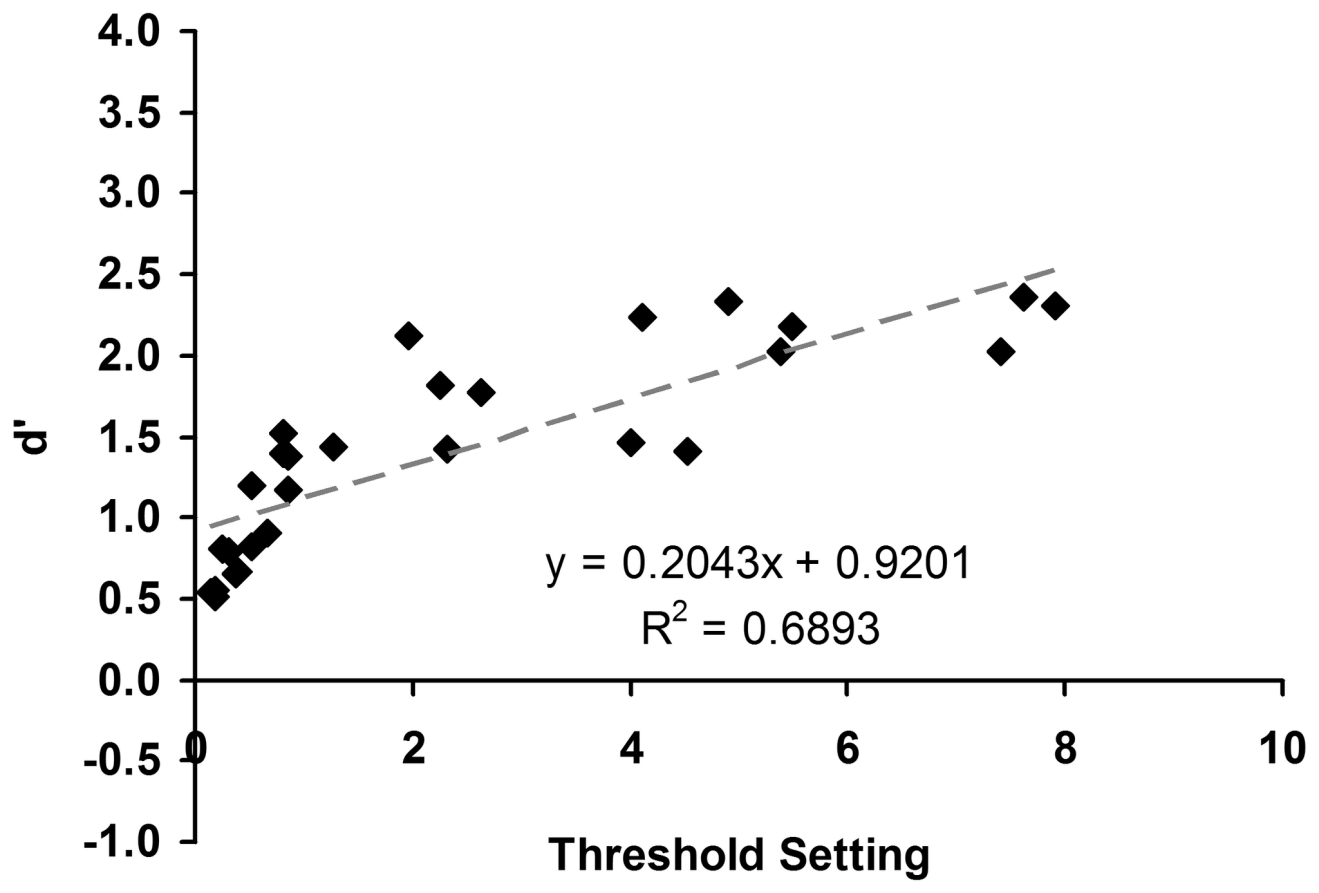
Individual d’ scores on pitch direction discrimination as a function of pitch direction threshold.

Correlations between average memory performance and pitch direction thresholds showed that those with higher thresholds tended to perform better (r(26) = 0.83, p < 0.05, see Figure 7), although this may be due to the higher performance of the amusic group. When these correlations were performed for the amusics and the controls alone, respectively, the correlations were significant [r(12) = 0.76, and 0.88 for the amusics and the controls, respectively, *p*s < 0.05].

Overall, the pitch direction retention data suggest that when a constant value (four semitones) is employed, amusics show poorer performance than the controls on the memory task. Moreover, while both groups improved at a similar rate as the discrimination was made easier during the silence condition, the amusic group showed a greater benefit than the controls as the discrimination was made easier when the interval was filled with distracting tones.

## Discussion and Conclusions

The goal of the current study was to examine whether or not difficulties with pitch discrimination affects pitch memory performance by testing individuals with congenital amusia. This may provide a reliable test case since congenital amusia is characterized by a deficit in the detection of changes in pitch, and recent studies have suggested there may be an associated pitch memory problem [28–32]. Consistent with previous findings, the current study demonstrated that individuals with congenital amusia have impaired performance on both a single-tone pitch retention task and a pitch direction retention task when a constant four semitone pitch distance between the standard and the comparison tones is used during different trials. However, the amusic group did not show impaired performance on these tasks when the perceptual difficulty of the discrimination was equated at the individual level. Moreover, as the discrimination between the standard and the comparison tones was made easier, memory performance increased at the same rate for both the amusic and control group. Combined, these findings suggest that the performance difference on pitch memory tasks that is found when the same stimuli are used for both the amusic and control groups may reflect differences between the groups in terms of the difficulty of the discrimination rather than reflecting a deficit in the amusics’ tonal memory system. These findings highlight the importance of equating for the difficulty of pitch discrimination when investigating pitch memory.

### The effect of the difficulty of pitch discrimination on pitch memory tasks

The current findings suggest that in pitch memory tasks there is a negative effect of discrimination difficulty on memory performance, which means differences in performance on a memory task may reflect differences in either memory or discrimination difficulty. Amusics were found to have higher pitch discrimination thresholds and higher pitch direction discrimination thresholds than the control group, reflecting the increased difficulty amusics have with these tasks relative to controls. Amusic thresholds from the two down one up staircase (approx. 70.7% detection threshold ) were nearly six times that of the controls (1.18 vs 0.20 semitones) for pitch discrimination and nearly four times that of the controls for the pitch direction discrimination (3.90 vs 1.01 semitones). Given these findings, when the same stimuli are used for both controls and amusics, such as the current four semitone conditions, then the stimuli are, by definition, closer in perceptual space to each other for the amusics than for the controls. This means the stimuli are perceptually more similar for the amusics than for the controls, resulting in a more difficult discrimination. As a consequence, the amusic group exhibited worse performance compared to the controls for both retention tasks.  Therefore, use of the same stimuli, which are more perceptually similar for the amusics than for the controls, may explain the difference in performance without need to suggest any deficit in a tonal memory system.  Overall, the current data are consistent with previous studies suggesting that pitch proximity is detrimental to performance on pitch memory tasks [4,11,12], and indicates that discrimination difficulty could account for the differences found on pitch memory performance.

This suggestion is further supported by the finding that when the stimuli were tailored to the individual participant’s 70.7% discrimination threshold, memory performance did not differ between amusics and controls. Both groups showed reduced performance on the 1x condition which used the threshold level stimuli. The staircase procedure should target a d’ of 1.76 [36] and both groups performed significantly worse than this at the 1x condition, indicating that there was signal loss, meaning memory trace degradation, over the retention period. However, there was no evidence that the amusic group suffered increased loss of information relative to the controls. As the discrimination between the standard and the comparison tones was made easier, by increasing the different trials as a function of the individual’s discrimination threshold, memory performance increased for both amusic and control groups at a similar rate. This is in keeping with results reported by Albouy et al. [28], suggesting that pitch memory performance increases with increasing interval changes for both amusic and control groups. 

Moreover, the current data may account for the effect of music expertise on pitch memory performance by suggesting that the pitch discrimination may be less difficult for musicians than nonmusicians and this difference in difficulty may explain the difference in pitch memory performance [3,4]. On the whole, the current findings indicate that the pitch discrimination difficulty, induced by pitch proximity, influences performance on pitch memory tasks specifically, and suggests that perceptual difficulty plays a prominent role on memory task performance in general.

### Implications for pitch memory in congenital amusia

The current data are consistent with previous studies [28–32], in that the amusics exhibited worse memory performance compared to controls when stimuli with a constant pitch distance of four semitones was employed for both of the retention tasks. In the past this reduced performance has been interpreted as evidence for pitch memory impairments associated with amusia. However, as a constant pitch distance is always much closer to the discrimination threshold for the amusic group than for the control group, it is argued here that this reduced performance reflects the difference in the perceptual difficulty of the task.

When the stimuli were tailored to the individual participant’s discrimination threshold, memory performance improved as the discrimination was made easier for both groups in the silence condition. However, amusics did not show any pitch retention deficits when the perceptual difficulty was equated in the current study. This differs from the study reported by Tillmann et al. [30], in which the authors intended to avoid the effect of perceptual deficits on pitch memory. Two explanations may account for the discrepancy. 

One explanation may be differences in how the perceptual difficulty has been equated. Although Tillmann et al. [30] intended to avoid the effect of perceptual deficits on pitch memory, they did not adjust the stimuli at the individual level. Specially, only three sets of stimuli with three sizes of pitch interval were employed: Set 1 (average intervals of 1.8 semitone), Set 2 (average intervals of 3.8 semitones), and Set 3 (average intervals of 5.4 semitones). While Set 1 was used for all controls whose thresholds ranged from 0.07 to 1.67 semitones, amusics used the three sets based upon their pitch thresholds, which ranged from 0.2 to 4 semitones: Set 1, 2, and 3 were used for amusics with thresholds below one semitone, between one and two semitones, and above two semitones, respectively. It is apparent that the difference in pitch discrimination difficulty between participants could still affect pitch memory performance since the difficulties were not entirely equated in Tillmann et al.’s previous study [30], as they were by using individually calculated pitch discrimination thresholds in the current study. It should be noted that if the four amusics with thresholds over 1 from the current study are dropped, none of the current results change. Therefore, given that the stimuli were not equated at the individual level in the previous study [30], the groups still differ in the difficulty of the pitch discrimination. 

A second explanation may reflect the difference between the stimuli used in the two studies. As noted above, the two tone pairs of the standard and comparison tones were not tonal in current study. Moreover, even for intervening tones, most of them did not correspond to semitones, and overall the stimuli in the current study were not tonal. However, tonal sequences were employed in study by Tillmann et al. [30]. It has been shown that tonal sequences facilitate memory performance not only for musicians [46], but also for nonmusicians [47]. In addition, expertise in other domains, such as chess, is known to improve memory performance for items that conform to the rules of activity compared to stimuli that do not [48]. Given that amusics fail to detect out-of-key tones [49], and as a group amusics listen to music much less frequently than the general population [50], it is reasonable to expect that by ensuring tonality of the stimuli in the previous study [30] the controls may have benefited in their memory performance more than the amusics. It should be noted that if the reduction in amusics’ performance is due, even in part, to reduced expertise with tonal stimuli then this is not really a deficit of the memory system per se but rather points to the argument that their underlying perceptual problems results in a lifestyle being one in which music plays a lesser role, and may even be actively avoided[50], and as a consequence their familiarity of musical structure (tonality) is reduced, which in turn makes the task more difficult. 

The current data show an effect of interference on memory performance for both the two groups suggesting higher memory performance in the silence condition than in the distractor condition. Consistent with the results reported by Williamson et al. [31], the effect of interference was not significantly larger in amusics relative to controls in the current study. This is inconsistent with the data reported by Gosselin et al. [29] in which there was an interaction between group and pitch distance indicating amusics showed larger interference effect for the pitch distance of 2 tones than those of 3 tones between the standard and the comparison tones, as compared with controls. As noted above, the same stimuli for amusics and controls were used in Gosselin et al. [29]. Therefore, the larger interference effect for the pitch distance of 2 tones may be due to pitch discrimination difficulties for amusics in the study by Gosselin et al [29]. 

The findings that pitch perceptual thresholds were not related to the single-tone pitch retention performance indicate the current threshold procedure is suited for equating the stimuli. However, with respect to the pitch direction retention, it appears that this is not the case. This may be due to the difference between an AXB task and a two alternative forced choice task. For pitch direction, the AXB threshold task is in all probability accomplished by the difference strategy [51], in which three pairs of tones are presented for pitch direction threshold in the current study. Within each pair, the time between tones is short. The memory task, however, presents two tones with a large delay between them. This changes the task to be effectively a two alternative forced choice task as one always knows there are two different stimuli and one just has to decide if the order was high – low or low –high [36]. The 2AFC task is known to be easier than the difference strategy [36], and so in this case, the memory test for pitch direction also includes switching to an easier discrimination.

The current study showed that amusics had better memory performance for pitch direction than controls when pitch direction perceptual difficulty was equated. Three explanations may account for the better performance in amusia. First, consistent with previous studies showing that amusics have more difficulty processing pitch direction than pitch change detection [18,32], the current data showed higher pitch direction threshold than pitch change detection thresholds in amusia. This indicates that pitch direction threshold may be harder for amusics, and consequently, amusics may pay more attention to the task at hand. When this is accompanied by a shift to an easier discrimination for the memory task, they may benefit more. Second, as noted above, in the pitch direction task, the time between the two tones is short during the AXB task while it is long during the memory phase. If part of the difficulty in making pitch direction decisions is not just hearing the difference between the tones, but also properly encoding the order of the tones, the threshold setting will reflect some combination of pitch and temporal order discrimination difficulty. By greatly increasing the temporal separation between the tones during the memory test it is possible this may benefit those higher thresholds more because it is possible their higher thresholds may, in part, be due to poor temporal order resolution. Finally, amusia is known to impact upon the perception of language tones as well [17,20–23], then it is possible that Mandarin speaking amusics practice at specifically remembering tonal direction changes, while the controls would be more tuned into the semantics or meaning only as the “sound” of the word would be easier for them to generate than for the amusics. Given this data, one might be tempted to suggest that amusia may lead to better memory for pitch direction changes, at least in those who speak a tonal language. While these are speculative explanations, the current finding further highlights the importance of equating pitch perceptual difficulty when investigating pitch memory for amusics. 

In summary, the current study demonstrates the effect that the difficulty of pitch discrimination has on pitch memory performance and how this complicates assessments of pitch memory in congenital amusia. However, we do not intend to imply that these results definitely prove that pitch memory is unaffected in amusia but rather we make the more modest suggestion that these data demonstrate the importance of equating for the known perceptual deficits associated with amusia before attempting to study memory performance. In order to demonstrate that amusics have an impairment in pitch memory, we recommend that future studies control for 1) the perceptual difficulty of the discriminations at the individual level rather than control the physical pitch difference of the stimuli and 2) avoid modification of the stimuli to conform to music rules as this will result in a stimuli set that is biased in favour of the control group, who may be considered expert music listeners relative to the amusic group. Finally, similar care must be taken to equate the discrimination difficulty of the stimuli before inferences about memory for other sound qualities, such as timbre [33], can be made. Indeed, these results demonstrate the importance of ensuring there are no perceptual or experience differences whenever comparing memory performance between any two groups. Discrimination sensitivity testing should be routine, and whenever possible, the memory stimuli should be equated for individual discrimination performance. Moreover, these complications increase when the stimuli to be employed are short musical phrases, for not only will such stimuli be perceptually more difficult for the amusics, but the differences in music exposure during everyday life, and therefore expertise, must also be considered when interpreting the results.
